# *Bifidobacterium* and the intestinal mucus layer

**DOI:** 10.20517/mrr.2023.37

**Published:** 2023-09-27

**Authors:** Alyssa Gutierrez, Brenton Pucket, Melinda A. Engevik

**Affiliations:** ^1^Department of Regenerative Medicine and Cell Biology, Medical University of South Carolina, Charleston, SC 29425, USA.; ^2^Department of Microbiology & Immunology, Medical University of South Carolina, Charleston, SC 29425, USA.

**Keywords:** Bifidobacterium, mucus, intestine, probiotic

## Abstract

*Bifidobacterium* species are integral members of the human gut microbiota and these microbes have significant interactions with the intestinal mucus layer. This review delves into *Bifidobacterium*-mucus dynamics, shedding light on the multifaceted nature of this relationship. We cover conserved features of *Bifidobacterium*-mucus interactions, such as mucus adhesion and positive regulation of goblet cell and mucus production, as well as species and strain-specific attributes of mucus degradation. For each interface, we explore the molecular mechanisms underlying these interactions and their potential implications for human health. Notably, we emphasize the ability of *Bifidobacterium* species to positively influence the mucus layer, shedding light on its potential as a mucin-builder and a therapeutic agent for diseases associated with disrupted mucus barriers. By elucidating the complex interplay between *Bifidobacterium* and intestinal mucus, we aim to contribute to a deeper understanding of the gut microbiota-host interface and pave the way for novel therapeutic strategies.

## INTRODUCTION

### Intestinal mucus

The intestine is continually exposed to a multitude of luminal antigens and bacterial components. To protect itself, the intestinal epithelium harbors specialized cells known as goblet cells, which synthesize and secrete mucus. The structure of intestinal mucus is intricately designed to form a protective barrier. The primary structural component of intestinal mucus is a gel-forming glycoprotein called MUC2. MUC2 is a large, heavily glycosylated protein that forms disulfide-bonded dimers. These dimers undergo further polymerization and crosslinking, resulting in the formation of a gel-like network that constitutes the mucus layer.

In addition to the gel-forming MUC2, intestinal mucus contains a diverse array of compounds that contribute to its composition and functionality. Mucus harbors Antimicrobial Peptides (AMPs): small cationic peptides that possess antimicrobial properties. AMPs in the mucus layer help to maintain the balance of microbial populations by inhibiting the growth of pathogenic bacteria and promoting the growth of beneficial commensal bacteria. Immunoglobulin A (IgA) antibodies are also abundant in the mucus layer of the gut. They are produced by specialized immune cells called plasma cells and secreted into the mucus, where they play a crucial role in immune defense by neutralizing pathogens, preventing their adherence to the intestinal epithelium, and promoting their clearance from the gut. In addition to MUC2, goblet cells also secrete trefoil factors, a family of small peptides that contribute to the maintenance of mucosal integrity and repair by promoting epithelial cell migration, enhancing wound healing, and providing protection against injury and inflammation. Other mucus-associated proteins, such as FCGBP, metalloenzyme CLCA1, ZG16, Lypd8, glycosaminoglycans, and chitinases, contribute to the structural organization, hydration, and stability of the mucus layer^[1]^. These compounds play various roles in shaping the mucus layer and modulating host-microbe interactions within the gut.

The structural organization of intestinal mucus is highly dynamic, exhibiting regional variations along the gastrointestinal tract. In the small intestine, the mucus layer is thinner and less firmly attached to the epithelium, allowing for efficient absorption of nutrients. In contrast, the mucus layer in the colon is thicker and firmly adheres to the epithelial surface, serving as a physical barrier that limits direct contact between luminal contents and the epithelium. In the colon, the mucus layer is stratified, consisting of two distinct regions: the inner mucus layer and the outer mucus layer. The inner mucus layer, also known as the firmly adherent mucus layer, is in direct contact with the intestinal epithelium. It is tightly packed with MUC2, forming a dense and organized matrix that provides a protective barrier against luminal contents. The outer mucus layer, also referred to as the loose mucus layer, is less compact and acts as a reservoir for commensal bacteria and other luminal components. This outer layer is more penetrable and allows for the establishment of symbiotic interactions between the gut microbiota and the host.

One feature of mucus that makes it amenable to microbe interactions is the structure of the mucin proteins. The MUC2 protein is extensively O-glycosylated with branched oligosaccharides^[2-7]^. O-glycans are attached at serine and threonine residues in the MUC2 protein and consist of core structures of α- and β-linked N-acetyl-glucosamine, N-acetyl-galactosamine, and galactose. The core structures are then elongated and generally modified by α-linked fucose, sialic acid, and sulfate residues^[4]^. Mucin glycoproteins serve as both an adhesion site and nutrient source for the resident gut microbes, providing an array of complex microbe-host interactions.

### Bifidobacteria and mucus

Among the bacteria found in the gut microbiota, *Bifidobacterium* species are known to reside within the intestinal mucus layer^[8-14]^ and exert multiple beneficial effects on the host^[15-20]^. Bifidobacteria are Gram-positive anaerobic bacteria from the phylum Actinobacteria that can have a rod or a distinctive bifid (i.e., Y) shape. There are currently 55 recognized species and subspecies of *Bifidobacterium*^[21-23]^. These species can be grouped into seven phylogenetic clusters: *B. longum*, *B. adolescentis*, *B. pseudolongum*, *B. boum*, *B. asteroides*, *B. pullorum*, and *B. bifidum*.

Bifidobacteria are predominant in the healthy breast-fed infant gut due to the presence of human milk oligosaccharides (HMOs), which these bacteria are adept at utilizing^[24-27]^. Studies have suggested that *Bifidobacterium* species make up ~80% of a breast-fed infant gut microbiota^[28-32]^. The benefits of *Bifidobacterium* strains are especially pronounced in early life, encompassing epithelial maturation, immune cell activation, and gut-brain-axis crosstalk^[33-39]^. Upon the introduction of solid food and weaning, the level of intestinal bifidobacteria continually decreases until adulthood, at which point bifidobacteria are maintained at a relative abundance of about ~10% throughout adult life^[31,40-42]^. In the elderly, the level of bifidobacteria further diminishes to about 0%-5% relative abundance^[42]^. This reduction in bifidobacteria levels in the elderly has been linked to age-related alterations in lifestyle and environment. Interestingly, this decline in *Bifidobacterium* abundance coincides with a simultaneous decrease in the thickness of intestinal mucus and an increase in its permeability^[43-45]^. It remains uncertain whether there is a direct link between decreased *Bifidobacterium* and decreased mucus, but this interesting observation suggests a relationship. Independent of age, *Bifidobacterium* species can be found in both the small intestine and colon, although they exhibit a higher abundance in the colon. Several *Bifidobacterium* species have been observed to interact with intestinal mucus, colonize the mucus layer, consume mucus glycans, and exert strain-specific modulatory effects on the mucus layer. This review covers the existing literature for the following *Bifidobacterium*-mucus interactions: (1) mucus adhesion; (2) mucin glycan degradation; (3) positive modulation of goblet cell cells; (4) goblet cell retention during inflammation; and (5) suppression of pro-inflammatory cytokines and production of anti-inflammatory IL-10.

## MUCUS ADHESION BY BIFIDOBACTERIUM SPECIES

Multiple studies have demonstrated the ability of *Bifidobacterium* species to adhere to mucus [Table 1]. *B. adolescentis*, *B. angulatum*, *B. bifidum*, *B. breve*, *B. catenulatum*, *B. infantis*, *B. longum*, *B. infantis*, *B. animalis* subsp. *lactis*, and *B. pseudocatenulatum* have all been shown to bind to mucus isolated from the stool of human infants and/or adults^[46-51]^. *B. bifidum*, *B. breve*, *B. animalis*, *B. animalis* subsp. *lactis*, *B. longum*, *B. longum* subsp. *infantis*, and *B. catenulatum* have also been demonstrated to bind to intestinal mucus isolated from the healthy part of resected colonic tissue^[52-58]^.

**Table 1:** Literature review of mucus adhering *Bifidobacterium* species and strains

**Bifidobacterium species**	**Mucus type**	**Ref.**
*B. animalis subsp. Bb12*	Human stool mucus	[41]
*Bifidobacterium 420*		
*Bifidobacterium BF1100*		
*Bifidobacterium 913*		
*B. adolescentis JCMI275T*	Human stool mucus	[46]
*B. adolescentis JCM7042*		
*B. adolescentis JCM7046*		
*B. angulatum JCM7096T*		
*B. animalis JCM 1190T*		
*B. animalis JCM 1253*		
*B. animalis JCM 7117*		
*B. animalis JCM 7124*		
*B. bifidum JCM 1254T*		
*B. bifidum JCM 1255*		
*B. bifidum JCM 7004*		
*B. breve JCM1192T*		
*B. breve JCM7016*		
*B. catenulatum ATCC 27675*		
*B. catenulatum JCM 7131T*		
*B. infantis JCM 1210*		
*B. infantis JCM 1222T*		
*B. infantis JCM 1272*		
*B. animalis* subsp*. BbI2*		
*B. lactis JCM 10140T*		
*B. longum JCM 127F*		
*B. longum JCM 7052*		
*B. longum JCM 7054*		
*B. pseudocatenulatum JCM 1200T*		
*B. animalis sbusp. lactis Bb12*	Human stool mucus	[47]
*B. adolescentis JCM 2701T*	Human stool mucus	[48]
*B. angulatum* ATCC *27678 T*		
*B. longum subsp. infantis JCM 1222 T*		
*B. pseudocatenulatum JCM 1200 T*		
*B. bifidum JCM 1255 T*		
*B. breve JCM 1192 T*		
*B. catenulatum JCM 1194 T*		
*B. longum subsp. longum JCM 1217 T*		
*B. animalis subsp. lactis Bb12*		
*B. bifidum TMC3115*		
*B. bifidum TMC3103*		
*B. bifidum TMC3104*		
*B. bifidum TMC3108*		
*B. bifidum TMC3110*		
*B. bifidum TMC3112*		
*B. bifidum TMC3116*		
*B. bifidum TMC3119*		
*B. bifidum TMC3120*		
*B. bifidum TMC3121*		
*B. bifidum TMC3122*		
*B. animalis subsp. lactis Bb12*	Human stool mucus	[49]
*B. lactis Bb12*	Human stool mucus	[50]
*B. lactis Bb12*	Human stool mucus	[51]
*B. longum BIF9s*	Colonic tissue mucus	[52]
*B. longum BIF12s*		
*B. longum BIF13s*		
*B. catenulatum BIF31s*		
*B. breve 99* (*DSM 13692*)	Colonic tissue mucus	[54]
*B. lactis Bb12* (*DSM 10140*)		
*B. breve 99* (*DSM 13692*)	Colonic tissue mucus	[55]
*B. bifidum M6*	Colonic tissue mucus	[56]
*B. bifidum A1*		
*B. infantis BIR-0304*	Colonic tissue mucus	[57]
*B. infantis BIR-0307*		
*B. infantis BIR-0312*		
*B. catenulatum BIR-0324*		
*B. bifidum BIR-0326*		
*B. infantis BIR-0349*		
*B. breve BIR-0350*		
*B. longum BIR-BPD1*		
*B. longum BIR-BPD3*		
*B. longum BIR-BPG1*		
*B. longum BIR-BPG4*		
*B. bifidum M6*	Colonic tissue mucus	[58]
*B. bifidum M6dCo*		
*B. bifidum PBT*		
*B. bifidum PBTdOx*		
*B. animalis IPLA 658*		
*B. animalis 658dOx*		
*B. bifidum A8*		
*B. bifidum A8dOx*		
*B. bifidum A1*		
*B. bifidum A1dOx*		
*B. longum NIZO B667*		
*B. longum B667dCo*		
*B. animalis IPLA 4549*		
*B. animalis 4549dCo*		
*B. animalis 4549dOx*		
*B. bifidum DSM20456*	Colonic tissue mucus, Caco-2 cells	[53]
*B. bifidum MIMBb75*		
*B. animalis subsp. lactis Bb12*	Porcine intestinal mucus	[71]
*B. dentium ATCC 27678*	Germ-free mouse cecal mucus, HT29-MTX cells	[18]
*B. longum subsp. infantis ATCC 15697*		
*B. longum subsp. longum ATCC 55813*		
*B. breve ATCC 15698*		
*B. longum BIF 53*	Porcine stomach mucus	[70]
*B. lactis Bb 12*		
*B. longum BB 536*		
*B. longum NCC 2705*		
*B. longum W 11*		
*B. longum SP 07/3*		
*B. longum NCIMB 8809*		
*B. longum ATCC 15707*		
*B. longum BIR 324*		
*B. longum BIF 53*		
*B. animalis subsp. lactis IPLA4549*	HT29-MTX cells	[60]
*B. animalis subsp. lactis 4549dOx*		
*B. animalis subsp. lactis A1*		
*B. animalis subsp. lactis A1dOx*		
*B. animalis subsp. lactis A1dOx-R1*		
*B. longum NB667*		
*B. longum 667Co*		
*B. animalis* subsp*. lactis CCDM 374*	Caco-2 cells, HT29-MTX cells	[61]
*B. breve 4*	Caco-2 cells, HT29-MTX cells	[62]
*B. breve 5*		
*B. breve 25*		
*B. longum 4*		
*B. longum 16*		
*B. longum 18*		
*B. longum 22*		
*B. bifidum 8*		
*B. bifidum 7*		
*B. infantis 1*		
*B. animalis IATA-A2*	Caco-2 cells, HT29-MTX cells	[64]
*B. bifidum IATA-ES2*		
*Bifidobacterium animalis subsp. lactis Bb12*		
*B. bifidum DSM 20082*	Caco-2 cells, HT29-MTX cells; rat cecal mucus	[65]
*B. breve DSM 20213*		
*B. longum DSM 20219*		
*B. animalis DSM 20104*		
*B. longum CSCC 5089*	Caco-2 cells	[63]
*B. bifidum DNG6*	Caco-2 cells	[66]
*B. lactis NCC362*	Caco-2 cells	[67]
*B. longum NCC 490*		
*B. adolescentis NCC251*		
*B. bifidum NCC 189*		
*B. breve MB226*		
*B. bifidum S16*		
*B. bifidum S17*		
*B. infantis E18*		
*B. adolescentis ATCC 15706*	Caco-2 cells	[68]
*B. adolescentis TMC 2704*		
*B. adolescentis TMC 2705*		
*B. animalis TMC 5101*		
*B. infantis TMC 2906*		
*B. infantis TMC 2908*		
*B. longum TMC 2607*		
*B. longum TMC 2608*		
*B. longum TMC 2609*		
*B. bifidum TMC 3101*		
*B. bifidum TMC 3108*		
*B. bifidum TMC 3115*		
*B. bifidum TMC 3116*		
*B. bifidum TMC 3117*		
*B. breve TMC 3207*		
*B. breve TMC 3217*		
*B. breve TMC 3218*		
*B. breve TMC 3219*		
*B. infantis ATCC 15697*	Glycan array	[86]

Interestingly, *Bifidobacterium animalis* subsp*. lactis* and unclassified *Bifidobacterium* species were shown to adhere well to mucus isolated from the feces of newborns, 2-month-old infants, 6-month-old infants, and adults (25 to 52 years), but had substantially lower adhesion to mucus derived from the feces of elderly individuals (74 to 93 years)^[41]^. It was also found that *B. animalis* subsp. *lactis* had diminished adhesion to mucus isolated during episodes of diarrhea^[50]^. These findings point to the integrity of mucus for adhesion.

In addition to human stool and tissue derived mucus, *B. dentium*, *B. bifidum*, *B. adolescentis*, *B. breve*, *B. pseudocatenulatum*, *B animalis* subsp. *lactis*, *B. longum*, and *B. infantis* have been shown to bind to human mucus-producing HT29-MTX, Caco-2, INT-407, and LS-174T cells^[53,59-69]^ as well as to cecal mucus from germ-free mice and rats^[18,65]^ [Table 1]. *B. adolescentis*, *B. angulatum*, *B. longum*, *B. infantis*, *B. pseudocatenulatum*, *B. bifidum*, *B. breve*, *B. catenulatum*, and *B. animalis* subsp. *lactis* were also found to bind to pig stomach mucus^[48,70]^, and *B. animalis* subsp. *lactis* was reported to bind to pig intestinal mucus^[71]^. In agreement with these findings, *Bifidobacterium* species were found to have widespread adhesion to mucin gels created with pig stomach mucus in a bioreactor model^[72]^. These studies indicate that mucus adhesion is widely conserved among *Bifidobacterium* species.

**Figure 1:**
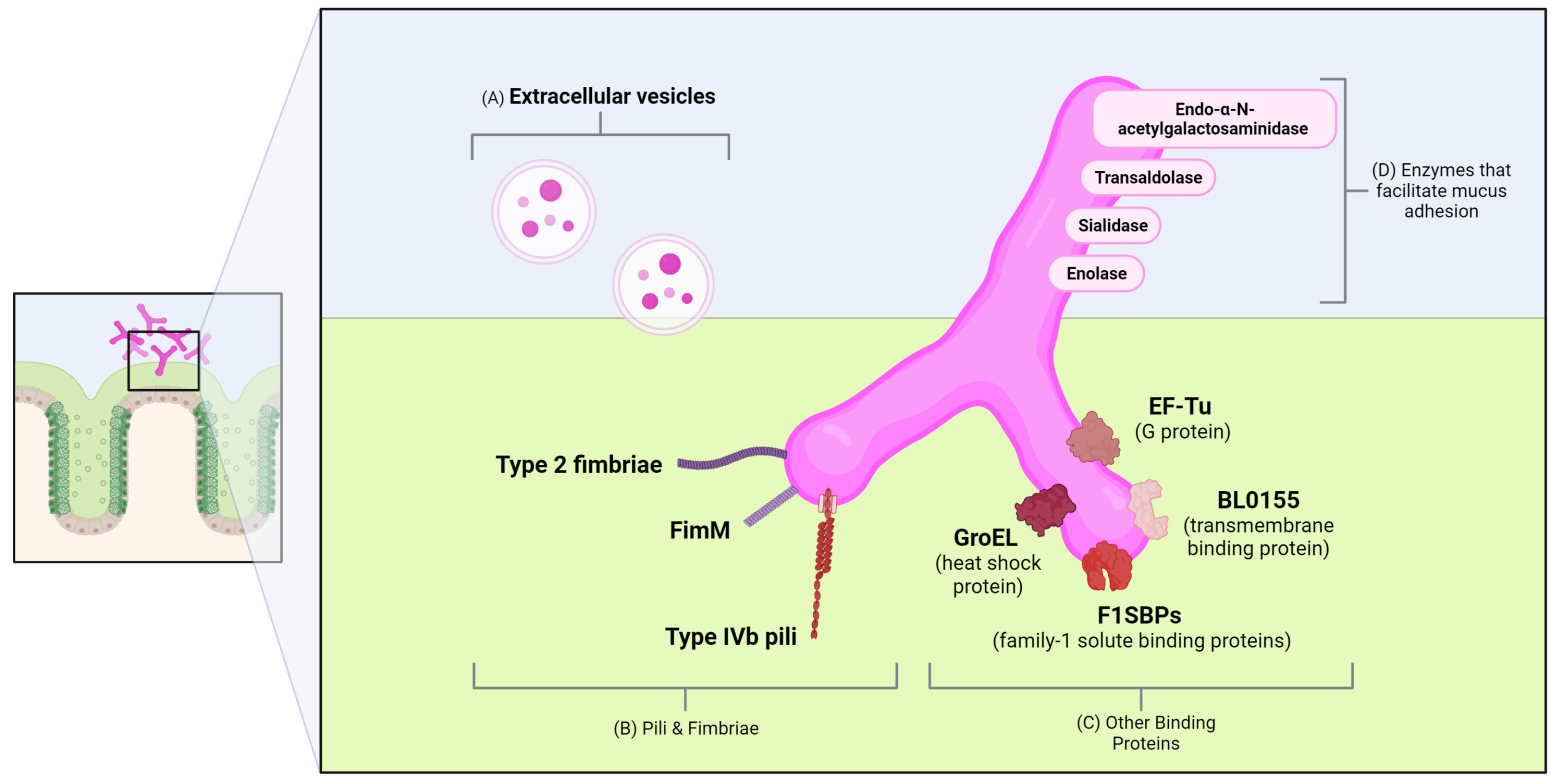
Schematic outlining examples of the various mechanisms by which *Bifidobacterium* adhere to mucus. (A) Extracellular vesicles released by *Bifidobacterium* can bind to mucus, and in turn, this binding can inhibit pathogen colonization; (B) *Bifidobacterium* possess a wide array of pili and fimbriae, including FimM and its homologs, type 2 fimbriae, and type IVb pili, which bind to mucus; (C) Other proteins such as F1SBPs (family-1 binding proteins), BL0155 (a type of ABC transport transmembrane protein), GroEL (a heat shock protein), and EF-Tu (Elongation Factor Tu) are involved in mucus binding; (D) Endo-α-N-acetylgalactosaminidase, transaldolase, sialidase, and enolase are enzymes that facilitate mucus adhesion.

The binding of *Bifidobacterium* to intestinal mucus is regulated by diverse adhesins [Figure 1]. *Bifidobacterium* species employ pili, surface adhesion proteins, moonlighting proteins, and other surface-anchored proteins to adhere to intestinal mucus [Table 2]^[73-75]^. For example, *B. bifidum* has several known mucin-binding partners. *B. bifidum* possesses two sortase-dependent pili that promote bacterial coaggregation and bind to mucus-producing Caco-2 cells^[76]^. Another study found that *B. bifidum* produces an extracellular sialidase that mediates adhesion to mucus via a conserved sialidase domain peptide that interacts with mucin carbohydrates^[77]^. Similar to *B. bifidum*, *B. longum* also expresses multiple mucus-binding proteins. *B. bifidum* and *B. longum* both have been shown to express extracellular transaldolases that function as an adhesin that is capable of binding mucin^[78,79]^. A recent study found that *B. longum* harbors 21 putative adhesion proteins^[75]^. Using an overexpression system in a heterologous host, it was found that FimM exhibited significant adhesion to mucus-producing LS174T goblet cells, and it was further found that mucin was one of the major adhesion receptors for the FimM protein^[75]^. Homologs of FimM were also identified in *B. bifidum*, *B. gallinarum*, and 23 other *B. longum* strains by sequence similarity analysis. Another study found that *B. longum* harbors a protein with high homology to type 2 glycoprotein-binding fimbriae that may mediate mucus adhesion^[80]^. *B. longum* additionally produces the moonlighting proteins EF-Tu and enolase, which indirectly promote adhesion to mucus-producing Caco-2 cells through interactions with host plasminogen^[81]^. Likewise, enolase plays the role of an adhesion factor in *B. lactis* Bl07^[82]^, and GroEL is another moonlighting protein that has been indicated as an adhesion factor for *B. animalis* subsp. *lactis*^[83]^.

**Table 2:** Literature review of mucus and cell binding adhesins in *Bifidobacterium* species and strains

***Bifidobacterium* species**	**Adhesin**	**Ref.**
*B. bifidum* PRL2010	Sortase-dependent pili	[76]
*B. bifidum* ATCC 15696	Extracellular sialidase	[77]
*B. bifidum* A8	Extracellular transaldolase	[79]
*B. longum* NCC2705	Extracellular transaldolase	[78]
*B. longum* BBMN68	Putative adhesion proteins	[75]
*B. longum* BBMN68	FimM	[75]
*B. bifidum* 85B	FimM homologs	[75]
*B. gallinarum* CACC 514	FimM homologs	[75]
*B. longum* NCC2705	Type 2 glycoprotein-binding fimbriae homolog	[80]
*B. longum* NCC2705	EF-Tu	[81]
*B. longum* NCC2705	Enolase	[81]
*B. animalis* subsp. *lactis* Bl07	Enolase	[82]
*B. animalis* subsp. *lactis* KLDS 2.0603	GroEL	[83]
*B. longum* VMKB44	Blap-1	[84]
*B. longum* JCM1217	Endo-α-N-acetylgalactosaminidase	[85]
*B. longum* subsp. *infantis* ATCC 15697	Family 1 of solute binding proteins (F1SBPs)	[86,87]
*B. breve* UCC2003	Type IVb pilus-type proteins	[82,83,88]
*B. longum* NCC2705	Extracellular vesicles	[89]

As another example of the various adhesins employed by *Bifidobacterium* species, *B. longum* was found to possess a 26-amino-acid peptide called Blap-1 that mediates adhesion to HT-29 cells. Interestingly, genomic analysis revealed that Blap-1 was an identical match to a site in a large extracellular transmembrane protein encoded by the BL0155 open reading frame of *B. longum* NCC2705^[84]^. Additionally, *B. longum* possesses an endo-α-N-acetylgalactosaminidase that contains binding sites specific to the protein core of mucin glycoproteins^[85]^. Furthermore, the genome of *B. longum* subsp. *infantis* encodes several family 1 of solute binding proteins (F1SBPs), and these proteins were shown to bind and transport mucin oligosaccharides^[86,87]^. In addition to *B. bifidum* and *B. longum*, *B. breve* has type IVb pilus-type proteins that facilitate colonization in the host gut^[82,83,88]^. Interestingly, it has also been shown that *B. longum* produces extracellular vesicles that export mucin-binding cytoplasmic proteins, and these proteins promote the adhesion of *B. longum* to mucus^[89]^. It has also been recently shown that the polyamine Spermidine significantly increased the adhesion of *B. bifidum* Bb12 to mucus isolated from healthy infants^[90]^, suggesting that secreted factors could also influence the adherence of *Bifidobacterium* to mucus. Together, these studies indicate that although multiple *Bifidobacterium* species can bind to mucus, the mechanisms of adhesion appear to be diverse, even among strains of the same species.

The structure of mucus likely dictates the consequences of mucus binding for *Bifidobacterium* species. In the small intestine, the mucus is loose and not attached to the epithelium. As a result, mucus adhesion likely does not promote persistent colonization of the small intestine. In contrast, in the colon, the mucus is highly organized and adhesion to colonic mucus most likely allows *Bifidobacterium* species to persist and colonize the colon. The adhesion of *Bifidobacterium* to colonic mucus is also thought to increase the transit time of the bacteria in the gut, thereby maximizing its beneficial properties^[91,92]^. It has also been shown that colonization of the mucus layer by *Bifidobacterium* species positively regulates goblet cells. These interactions are all viewed as beneficial for the host. As a result of these positive attributes, the ability to adhere to human intestinal mucus is a commonly employed criterion for the selection of probiotic organisms^[75,93,94]^.

The binding of *Bifidobacterium* to intestinal mucus extends beyond a mere physical attachment; it serves as a gateway for host-microbe crosstalk. By positioning themselves within the mucus, *Bifidobacterium* strains gain proximity to host cells, enabling the effective delivery of health-promoting molecules, metabolites, and signaling compounds^[18,95,96]^. Furthermore, the presence of *Bifidobacterium* within the mucus layer influences the spatial organization and composition of the gut microbiota, thereby impacting the overall microbial ecosystem. In several studies, the ability to bind to the mucus layer allowed *Bifidobacterium* species to create a niche and exclude pathogens^[54,56,57,62,64,92,97]^. One study found that a probiotic containing *Bifidobacterium* could inhibit pathogenic colonization of *Escherichia coli*, and this protective effect was dependent on MUC2 expression by Caco-2 cells^[98]^. This data suggests that mucus adhesion is critical for excluding pathogens. In addition to excluding pathogens, *Bifidobacterium* species likely have synergistic interactions with other commensal microbes in the mucus layer. *Bifidobacterium* has been shown to cross-fed commensal *Eubacterium rectale*^[99]^, *E. hallii*^[100,101]^, and *Faecalibacterium prausnitzii*^[102]^. In each of these scenarios, *Bifidobacterium*-commensal co-cultures generated elevated levels of butyrate, a beneficial short-chain fatty acid, compared to the mono-cultures. The literature clearly indicates that *Bifidobacterium* species readily bind to mucus, and this mucus adhesion likely sets the stage for a range of beneficial effects on both the host and the gut microbial community.

## MUCUS DEGRADATION BY *BIFIDOBACTERIUM* SPECIES

In addition to serving as a binding site for bacteria, mucus can act as a nutrient source. The mucin protein is heavily O-glycosylated and has multiple structures of repeating α- and β-linked N-acetyl-galactosamine (GalNAc), N-acetyl-glucosamine (GlcNAc), and galactose (Gal) residues, terminated with α-linked fucose (Fuc), and sialic acid (Neu5Ac) residues^[103]^. Mucus-degrading bacteria harbor specific glycosyl hydrolases (GHs) that enzymatically degrade mucin glycans^[3,4,103-106]^. After cleavage, the released glycan oligosaccharides can feed the bacteria or other microbes in the vicinity^[3,107]^. In order to degrade mucin glycans, intestinal bacteria must possess GH33 sialidases (also known as neuraminidases), which cleave terminal sialic acid residues. For efficient glycan cleavage, bacteria can also generate GH29 or GH95 to remove fucose residues. Once the terminal sugars are removed, the underlying GalNAc, GlcNAc, and galactose residues can be removed. Bacteria can have GH101 or GH129 to remove GalNAc, GH84, GH85, GH89, or GH20 to remove GlcNAc, or GH2, GH35, GH42, and GH98 to remove galactose residues. Some bacteria also encode for GH16, endo-acting O-glycanases that remove larger glycan structures. A recent genome analysis confirmed that *B. bifidum* harbored the largest repertoire of mucus-degrading GHs among the *Bifidobacterium* species^[103]^. All *B. bifidum* genomes had GH33, GH29, GH95, GH20, GH2, GH42, GH101, GH129, GH89, and GH84^[103]^, suggesting that this species was capable of cleaving sialic acid, fucose, GalNAc, GlcNAc, and galactose from mucus glycans. *B. breve*, *B. longum*, and *B. scardovii* were also found to possess multiple mucus-associated GHs. This finding is consistent with other genome studies and *in vitro* studies, which report that *B. bifidum*, *B. longum*, and *B. breve* can degrade mucus^[19,100,103,108-112]^. In contrast, *B. adolescentis*, *B. angulatum*, *B. animalis*, *B. dentium*, *B. pseudolongum*, and *B. thermophilum* possessed few mucus-associated GHs^[103]^. *In vitro* work confirmed that *B. dentium* and *B. angulatum* were unable to grow on pig colonic mucus as the sole carbon source^[103]^. Separate studies have also found that *B. animalis* subsp. *lactis* and *B. pseudolongum* do not degrade mucus^[100,113-115]^. These studies suggest that, unlike mucus adhesion, mucus degradation is not conserved in *Bifidobacterium* species^[103]^.

Mucin degradation is considered to be a normal process of intestinal mucus turn-over^[116]^ and begins within the first few months of life^[117,118]^. Infants are commonly colonized with mucin-degrading *B. bifidum*, *B. longum* subsp. *infantis*, and *B. breve*^[29,30,118,119]^, as well as *Akkermansia muciniphila* and *Bacteroides* species^[116]^. Interestingly, breast-fed babies that are dominated by *Bifidobacterium* species exhibit a delay in the mucin degradation profile as compared with babies fed with formula milk^[118]^. Consistent with this notion, Karav *et al.* found that supplementation of *B. longum* subsp. *infantis* EVC001 to healthy breast-fed infants significantly reduced the proportion of free colonic mucin-derived O-glycans in the total glycan pool to 1.87% compared to 37.68% in the control infants who did not receive supplemented *B. longum*^[120]^. The level of freed mucin-derived O-glycans was negatively correlated with populations of *Bifidobacteriaceae*, indicating that mucus degradation was not occurring at the same level in *B. longum* supplemented infants^[120]^. Along the same lines, genes involved in mucus-degrading pathways, particularly in carbohydrate metabolism, in *Bifidobacterium* species were found to be expressed to a greater degree in formula-fed infants than in breast-fed infants^[121]^. It has been speculated that HMOs, which are similar to mucus in some of the glycan structures^[121,122]^, or other mucin-like glycoproteins present in breast milk, may compete with intestinal mucus as a substrate^[118]^.

In addition to being found in infants, mucus-degrading *Bifidobacterium* species are present in adults and have been linked to the suppression of detrimental mucus degradation. One example of excessive mucus degradation that may be prevented by Bifidobacteria is in the context of a Westernized diet, a diet characterized by low fiber but high fat and sugar. It has been demonstrated in mice harboring defined microbial communities that consuming a Westernized diet leads to an expansion of mucin-degrading bacteria such as *Akkermansia muciniphila* and *Bacteroides caccae*, and this shift enables the bacterial community to target the mucus layer for digestion in lieu of dietary fibers^[123]^. In a model with complex native gut microbiota, mice fed a Westernized diet similarly exhibited an expansion of *Akkermansia* and a corresponding decrease in *Bifidobacterium* species^[124]^ and increased susceptibility to pathogens and inflammation. In this setting, the addition of *B. longum* NCC 2705 or the prebiotic inulin resulted in elevated levels of endogenous *Bifidobacterium* species, reduced mucus degradation, and restored the mucus barrier. In a similar vein, *B. bifidum* G9-1 was shown to protect against mucus degradation by *A. muciniphila* following small intestine injury caused by a proton pump inhibitor and aspirin^[125]^. Another study found that the administration of *B. pseudolongum* Patronus increased mucosal thickness in rats and decreased the levels of *A. muciniphila*^[126]^. These data suggest that mucus degradation by *Bifidobacterium* species is not detrimental to the host and that *Bifidobacterium* species keep mucus degradation in check.

## MUCUS MODULATION BY *BIFIDOBACTERIUM* SPECIES

### Modulation of mucus by Bifidobacteria in homeostasis

**Figure 2:**
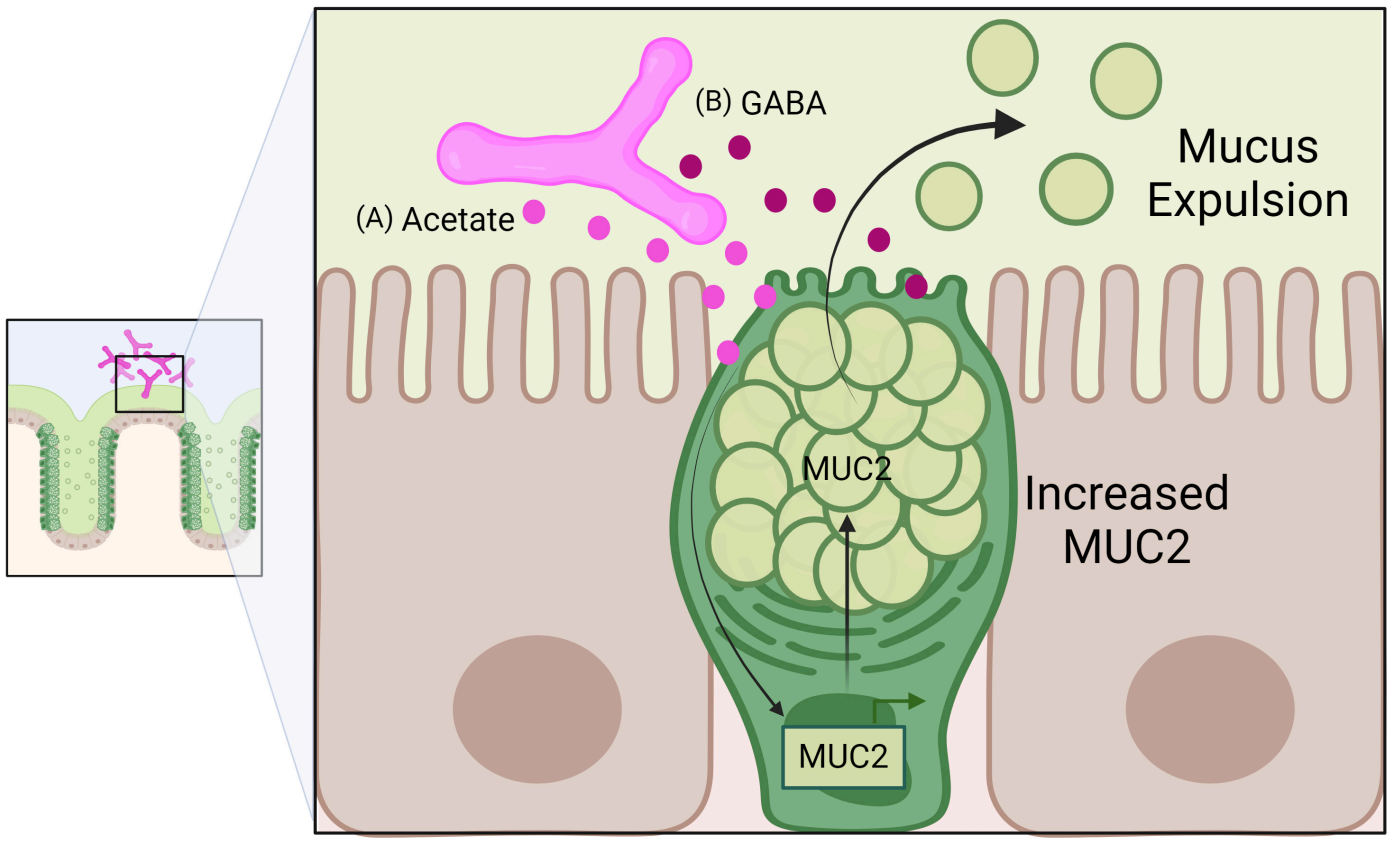
Representative diagram of *Bifidobacterium-goblet* cell interactions. (A) *Bifidobacterium* species can generate acetate, which can elevate MUC2 expression and protein; (B) *Bifidobacterium* species can also generate varying levels of GABA, which can activate autophagy-driven expulsion of mucus. Through these mechanisms, Bifidobacteria are speculated to positively regulate goblet cells. GABA: *B. dentium*-generated gamma-aminobutyric acid.

Although some bifidobacteria have mucolytic properties, they generally have an overall positive net effect in regulating intestinal mucus. Several studies have found that *Bifidobacterium* species elevate mucus levels *in vitro* and *in vivo* [Table 3 and Figure 2]. *In vitro*, *B. infantis*, *B. breve*, *B. longum* and a probiotic cocktail containing these microbes and others (VSL#3) was found to stimulate mucus secretion in human mucus-producing LS174T cells^[127]^. The probiotic cocktail was also found to increase MUC2 expression and secretion in rat colonic loops^[127]^. In another study, *B. dentium* was reported to increase MUC2 in human mucus-producing T84 cells^[18]^. Short-chain fatty acids (SCFA) have been demonstrated to increase MUC2 expression^[128]^, and *Bifidobacterium* species are known to produce high levels of SCFA acetate. The application of acetate was likewise able to increase MUC2 gene and protein levels in T84 cells^[18]^. *In vivo*, *B. dentium* was found to colonize germ-free mice, elevate intestinal acetate levels, and increase MUC2 at the gene and protein levels^[18]^. An elevated number of goblet cells and goblet cell-specific genes were observed in *B. dentium* mono-associated mice, as well as increased mucin glycosylation^[18]^. In this model, it was speculated that *B. dentium*-generated gamma-aminobutyric acid (GABA) was able to activate autophagy and calcium signaling to stimulate the release of mucus from goblet cells and bolster the mucus barrier^[18]^. In addition to B*. dentium*, *B. bifidum* and *B. longum* colonize germ-free mice and increase intestinal mucin glycoproteins^[129,130]^. These studies using mono-associated gnotobiotic animals provide very powerful evidence that *B. dentium*, *B. bifidum* and *B. longum* can modulate goblet cell function and increase mucus production. In mice with complex gut microbiota, *B. breve* supplementation led to 3,996 upregulated and 465 downregulated genes in supplemented neonatal mice relative to the untreated group^[35]^. Upregulated genes in the neonatal mice encoded multiple mucus layer-associated proteins such as MUC2. These data suggest that *B. breve* in early life modulates goblet cells. In adult mice, administration of a probiotic cocktail containing *B. breve* also increased the number of goblet cells per crypt and increased the production of mucus compared with controls^[131]^. Collectively, these data indicate that *Bifidobacterium* strains influence goblet cell function and mucus production.

**Table 3:** Literature review of the positive effects of Bifidobacteria on mucus expression, mucin levels and mucus expulsion

***Bifidobacterium* species**	**Finding**	**Experimental model**	**Ref.**
*B. infantis*	Increased mucus secretion	LS174T cells	[127]
*B. breve*	Increased mucus secretion	LS174T cells	[127]
*B. longum*	Increased mucus secretion	LS174T cells	[127]
*VSL#3*	Increased mucus secretion	LS174T cells	[127]
*VSL#3*	Increased MUC2 expression and secretion	Rat colonic loops	[127]
*B. dentium* ATCC 27678	Increased mucus expression and secretion	T84 cells	[18]
*B. dentium* ATCC 27678	Increased MUC2 expression and mucus levels	Adult gnotobiotic mice	[18]
*B. bifidum* FPLC AA22	Increased mucus levels	Adult gnotobiotic mice	[130]
*B. longum* FPLC 117	Increased mucus levels	Adult gnotobiotic mice	[129]
*B. breve* UCC2003	Increased MUC2 expression	Neonatal conventional mice	[35]
*B. breve* (probiotic cocktail)	Increased goblet cells per crypt and increased mucus levels	Adult conventional mice	[131]

### Modulation of mucus by Bifidobacteria in inflammation and infectious diseases

**Figure 3:**
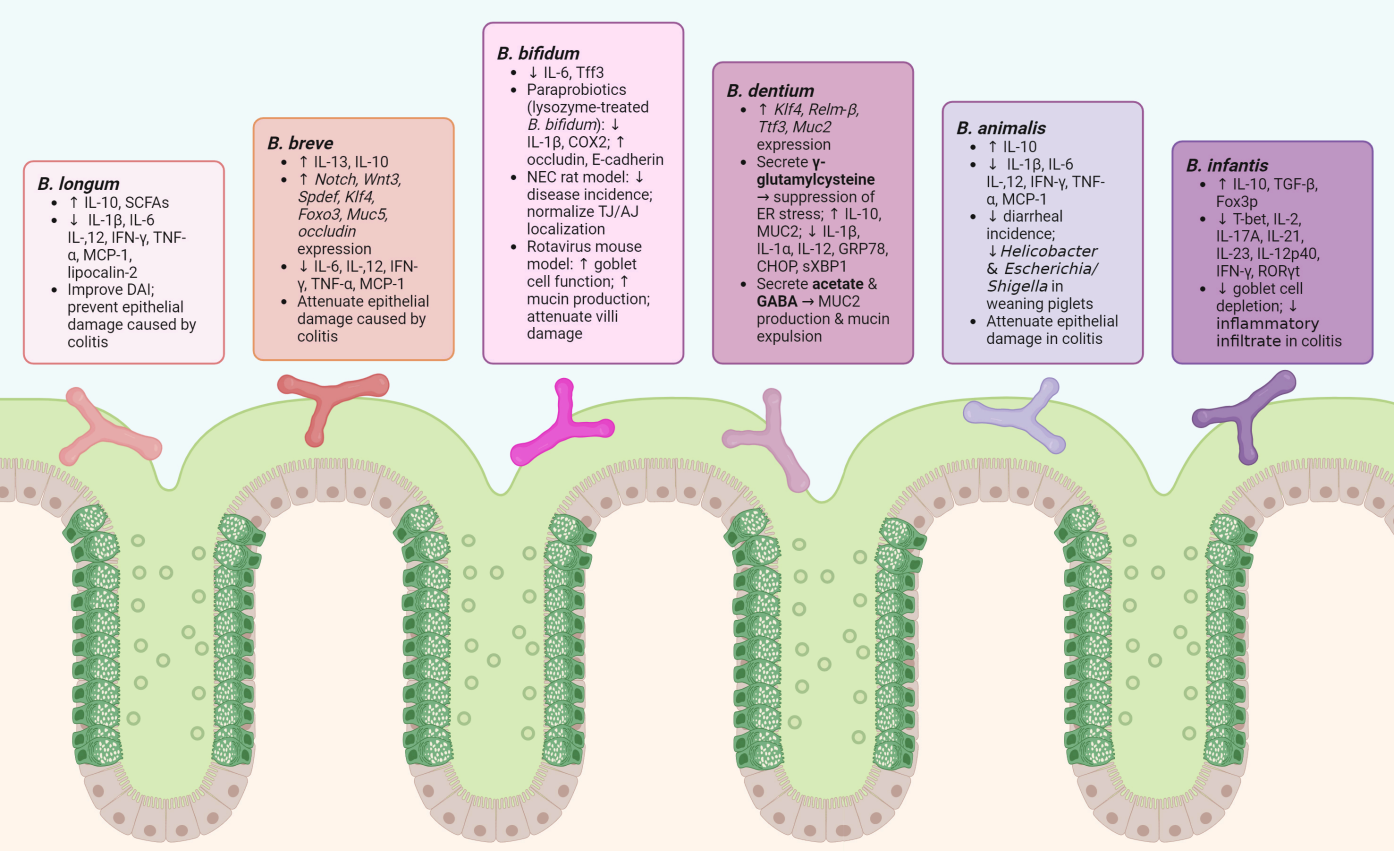
Diagram outlining the major beneficial effects, especially in improving goblet cell function and in reducing inflammation, per *Bifidobacterium* species in disease models. GABA: *B. dentium*-generated gamma-aminobutyric acid; NEC: necrotizing enterocolitis; SCFAs: short-chain fatty acids.

There is a wide array of data that demonstrate the substantial benefits of *Bifidobacterium* in the context of disease. Colitis is one of the most frequently investigated intestinal diseases, and a variety of *Bifidobacterium* species have exhibited the ability to alleviate major complications of colitis. In general, *Bifidobacteriu*m species have been shown to (1) limit inflammation-associated goblet cell and mucus depletion and MUC2 and (2) reduce pro-inflammatory cytokines [Figure 3, Tables 4 and 5].

**Table 4:** Literature review of strain-specific effects of *Bifidobacterium* species on mucus modulation in the context of inflammation or infectious disease

***Bifidobacterium* species**	**Finding**	**Intestinal site**	**Experimental model**	**Ref.**
*B. bifidum* FL-276.1	Increased MUC2, improved mucus, reduce colitis	Colon	DSS-colitis	[167]
*B. bifidum* FL-228.1	Increased MUC2, improved mucus, reduce colitis	Colon	DSS-colitis	[167]
*B. bifidum* BGN4	Improved mucus, reduce colitis	Colon	DSS-colitis	[169]
*B. longum* subsp. *longum* YS108R	Increased MUC2, improved mucus, reduce colitis	Colon	DSS-colitis	[170]
*B. longum* Bif10	Increased MUC2, improved mucus, reduce colitis	Colon	DSS-colitis	[172]
*B. breve* Bif11	Increased MUC2, improved mucus, reduce colitis	Colon	DSS-colitis	[172]
*B. breve* CBT BR3	Improved mucus, reduce colitis	Colon	DSS-colitis	[171]
*B. animalis* subsp. *lactis* A6	Improved mucus, reduce colitis	Colon	DSS-colitis	[168]
*B. infantis* GMCC0460.1	Improved mucus, reduce colitis	Colon	DSS-colitis	[173]
*B. infantis* 2017012	Improved mucus, reduce colitis	Colon	DSS-colitis	[177]
*B. infantis* unclassified strain	Improved mucus, reduce colitis	Colon	DSS-colitis	[177]
*B. breve* H4-2	Improved mucus, reduce colitis	Colon	DSS-colitis	[175]
*B. breve* H9-3	Improved mucus, reduce colitis	Colon	DSS-colitis	[175]
*B. lactis* BL-99	Improved mucus, reduce colitis	Colon	Zebrafish colitis model	[178]
*B. dentium* ATCC 27678	Increased MUC2, improved mucus, reduce colitis	Colon	TNBS-colitis	[96]
*B. infantis* unclassified strain	Improved mucus, reduce colitis	Colon	TNBS-colitis	[179]
*B. longum* Bar 33	Improved mucus, reduce colitis	Colon	TNBS-colitis	[177]
B. animalis subsp. lactis CNCM-I2494	Improved mucus, reduce colitis	Colon	DNBS-colitis	[180]
*B. bifidum* E3	Increased MUC2, improved mucus	Small intestine	LPS-induced injury	[181]
*B. infantis* E4	Increased MUC2, improved mucus	Small intestine	LPS-induced injury	[181]
*B. lactis* BB12	Increased MUC2, improved mucus	Small intestine	LPS-induced injury	[181]
*B. bifidum* OLB637	Increased mucin expression	Small intestine	Rat model of NEC	[182]
*B. bifidum* G9-1	Increased MUC2, improved mucus	Small intestine	Rotavirus mouse model	[186]
*B. infantis P*CM	Improved mucus	Small intestine	Cronobacter sakazakii mouse model	[187]

**Table 5:** Literature review of strain-specific effects of *Bifidobacterium* species on immune modulation in the context of inflammation

***Bifidobacterium* species**	**Finding**	**Body site**	**Experimental model**	**Ref.**
*B. infantis*	Reduced pro-inflammatory cytokines & increased IL-10	Colon	TNBS colitis	[177]
*B. breve* CBT BR3	Reduced pro-inflammatory cytokines & increased IL-10	Colon	TNBS colitis	[171]
*B. longum and B. animalis* (probiotic cocktail)	Reduced pro-inflammatory cytokines & increased IL-10	Colon	TNBS colitis	[179]
*B. dentium* ATCC 27678	Reduced pro-inflammatory cytokines & increased IL-10	Serum and colon	TNBS colitis	[96]
*Bifidobacterium animalis* subspecies *lactis CNCM-I2494*	Reduced pro-inflammatory cytokines & increased IL-10	Colon and T cells	DNBS colitis	[180]
*B. longum* Bif10	Reduced pro-inflammatory cytokines	Serum and colon	DSS colitis	[172]
*B. breve* Bif11	Reduced pro-inflammatory cytokines	Serum and colon	DSS colitis	[172]
*B. longum* Bif16	Reduced pro-inflammatory cytokines	Serum and colon	DSS colitis	[172]

Colitis-inducing compounds are known to activate ER stress^[132-135]^, and ER stress has been linked to intestinal inflammation in multiple animal models^[136-140]^. Goblet cells are particularly sensitive to ER stress since producing and folding MUC2 is a complex process^[141,142]^. It has been speculated that modulation of goblet cell ER stress by *Bifidobacterium* species may represent a key pathway by which bifidobacteria promote intestinal health. In mucus-producing Caco-2 cells, the application of live *B. breve* YIT 12272 and *B. adolescentis* YIT 4011T alleviated tunicamycin-induced ER stress^[143]^. In another study using mucus-producing T84 cells, it was shown that *B. dentium* ATCC 27678-secreted metabolites could also suppress tunicamycin- or thapsigarin-induced ER stress^[96]^. Analysis of the *B. dentium* metabolites revealed that this strain generated substantial levels of γ-glutamylcysteine, a compound that can be converted into the powerful antioxidant glutathione and suppress oxidative and ER stress^[132,134,144-148]^. *B. dentium* metabolites harboring γ-glutamylcysteine and application of commmerically available γ-glutamylcysteine both elevated glutathione, suppressed inflammatory NF-κB activation, reduced IL-8 secretion, and attenuated the induction of the unfolded protein response (UPR) genes GRP78, CHOP, and sXBP1 in T84 cells and TNBS-treated mice^[96]^. These data suggest that *Bifidobacterium* species can reduce goblet cell ER stress.

When goblet cells undergo ER stress, they are unable to adequately synthesize and secrete MUC2, leading to a reduction in goblet cell number and a thinning of the intestinal mucus layer. Several animal models have shown that goblet cell ER stress or loss of mucus leads to intestinal inflammation (*Winnie*, MUC2*^-/-^*, AGR2*^-/-^*, glycan deficiency, *etc*.)^[149-154]^. These animal model phenotypes closely resemble the intestinal issues observed in inflammatory bowel disease (IBD) patients, particularly in ulcerative colitis patients^[139,155-158]^. Ulcerative colitis patients have decreased goblet cell numbers, truncated mucin glycosylation, reduced mucus layer thickness, and limited mucus integrity^[138,156-161]^. Loss of both the thickness and integrity of the mucus layer is thought to promote bacterial-epithelial interactions and drive inflammation^[162-166]^.

Several studies have found that *Bifidobacterium* species can limit the reduction of goblet cells and improve the mucus barrier in the setting of chemically induced intestinal inflammation [Table 4]. For example, *B. bifidum*, *B. longum*, *B. longum* subsp. *longum*, *B. breve*, and *B. animalis* subsp. *lactis* were shown to increase MUC2, improve the mucus barrier, and ameliorate DSS-induced colitis^[167-173]^. A probiotic mixture containing *B. infantis* was also shown to enhance the mucus barrier in DSS-treated mice^[174]^. *B. infantis* and *B. breve* were likewise found to limit the reduction of goblet cells in DSS models^[175-177]^, and *B. lactis* was found to improve goblet cell counts in a zebrafish model of intestinal inflammation^[178]^. Along the same lines, *B. dentium* was also shown to increase MUC2, limit goblet cell reduction, and improve the mucus layer in a TNBS-induced model of colitis^[96]^. *B. infantis* and *B. longum* were also found to improve goblet cell numbers in TNBS-induced colitis^[177,179]^, while *B. animalis* subsp. *lactis* restored goblet cell populations in dinitrobenzene sulfonicacid (DNBS)-challenged mice^[180]^. These studies indicate that *Bifidobacterium* species can reduce goblet cell loss and mucus depletion in the setting of TNBS and DNBS-induced colitis.

*Bifidobacterium* species also have positive roles in modulating mucus in other inflammatory models. For example, *B. bifidum*, *B. infantis*, and *B. lactis* increased MUC2 in the small intestine during LPS-induced injury^[181]^. In a rat model of necrotizing enterocolitis (NEC), *B. bifidum* was shown to increase mucin and TFF3 expression and decrease the disease severity^[182]^. *B. longum* EVC001 and *B. infantis* BB-02 also decreased NEC occurrence in animals^[183,184]^. Even more promising is a double-blind, randomized, controlled study of very-low-birth-weight preterm infants, in which a combination of *B. breve* strain Yakult and *L. casei* strain Shirota completely prevented the occurrence of NEC in the intervention group, whereas 3.5% of the cases developed NEC in control without probiotics^[185]^. The mechanism by which *Bifidobacterium* confers its benefits in NEC is not fully understood but may be similar to colitis involving the mucus layer, intestinal permeability, and inflammation.

Rotavirus gastroenteritis is another disease where *Bifidobacterium* species have been shown to beneficially modulate the mucus layer. *B. bifidum* G9-1 was shown to increase MUC2, normalize mucin-positive goblet cells in the small intestine, and reduce the incidence, diarrheal scores, and intestinal damage in the supplemented group with rotavirus compared to the control group with rotavirus alone^[186]^. *B. infantis* PCM has also been shown to maintain goblet cells and reduce epithelial damage in the small intestine of mice infected with the pathogen *Cronobacter sakazakii*^[187]^. These data demonstrate that goblet cells and mucin production are also beneficially influenced by bifidobacteria in the small and large intestines in multiple inflammatory models.

Pro-inflammatory cytokines have been shown to negatively regulate goblet cells, while anti-inflammatory compounds such as IL-10 are known to alleviate ER stress and enhance goblet cell function. Another pathway by which *Bifidobacterium* species positively modulate goblet cells is through the modulation of intestinal cytokines [Table 5]. In TNBS-induced colitis mouse models, supplementation of *B. infantis*, *B. breve*, and probiotic cocktail mixes that included *B. longum* Bar 33 and *B. animalis* subsp. *lactis* Bar 30 resulted in reduced levels of several pro-inflammatory cytokines, e.g., IL-2, IL-1β, IL-13, IL-12p40, IL-17A, IL-21, IL-23, IFN-γ, TNF-α, and MCP-1, relative to the untreated TNBS groups^[171,177,179]^. These strains additionally led to rises in the anti-inflammatory cytokine IL-10^[171,177,179]^. Similarly, *B. dentium* reduced serum IFN-γ, IL-1α, IL-1β, IL-12, and TNF-α with a concomitant increase in IL-10 in comparison to the TNBS control mice^[96]^. Another study using DSS revealed that *B. breve* and *B. longum* lowered both systemic and colonic levels of TNF-α, IL-1β, and IL-6^[172]^. These studies suggest that in addition to directly modulating goblet cells through metabolites and suppression of ER stress, *Bifidobacterium* strains may be indirectly modulating goblet cell function via immune regulation.

## OVERALL EFFECTS OF BIFIDOBACTERIUM-MUCUS INTERACTIONS ON THE HOST

The literature suggests that the intestinal mucus layer plays a crucial role in the interaction of *Bifidobacterium* species with the host. It appears that the majority of *Bifidobacterium* species bind to intestinal mucus and establish a unique niche that affords them an advantageous position for their beneficial activities. Within the mucus layer, some *Bifidobacterium* species can degrade mucus, while others must rely on other nutrient sources. In the mucus layer, *Bifidobacterium* species likely perform the following functions: (1) exclude pathogens; (2) cross-fed commensal bacteria; (3) limit excessive mucus degradation; (4) secrete compounds such as acetate, which elevate MUC2 expression and increase mucus production; (5) reduce goblet cell ER stress; (6) limit inflammation- and infection-driven goblet cell loss; (7) suppress pro-inflammatory cytokines; and (8) increase anti-inflammatory pro-goblet cell IL-10.

The literature points to the capacity for *Bifidobacterium* species to beneficially modulate goblet cell number and function, thereby regulating the mucus layer and intestinal barrier function. This modulation of the goblet cells by *Bifidobacterium* is likely even more important during the setting of infection and inflammation. Through these interactions, *Bifidobacterium* species facilitate a dynamic interplay that contributes to gut homeostasis and overall host health.

## LIMITATIONS AND GAPS IN THE FIELD

While these findings are compelling, there are still several gaps in knowledge. First, it is unclear which *Bifidobacterium* strains are the most effective at positively regulating goblet cell function. Very few studies have performed head-to-head comparisons of different *Bifidobacterium* strains and studies vary in terms of mouse strain (C57B6/J, BALBc, Swiss Webster, *etc.*), colonization status (mono-association, gnobotioic with defined communities, conventional, *etc.*), and challenge (TNBS, DSS, DNBS, LPS, infection *etc.*). These variables make it difficult to tease out the nuances between strains and effects. Second, the metabolites that drive goblet cell-specific attributes of *Bifidobacterium* are not well characterized. It is well documented that *Bifidobacterium* species can generate acetate and this SCFA can elevate MUC2 levels, but it is likely that other metabolites also stimulate MUC2. In addition to modulating MUC2 levels, *Bifidobacterium* species can influence goblet cells in other ways, such as suppressing ER stress, promoting autophagy, and stimulating mucus expulsion. Likewise, it is not clear how bifidobacteria members regulate IL-10 production, which could indirectly affect goblet cell homeostasis. These pathways need to be explored with multiple *Bifidobacterium* strains.

The advent of intestinal organoids is a promising new technology to address *Bifidobacteri*um-goblet cell interactions. This model maintains segment specificity, is not immortalized, and is not cancer-derived. Importantly, intestinal organoids harbor MUC2-positive goblet cells and have been previously used to examine bacterial-host interactions, including *Bifidobacterium*^[95,188-190]^. We anticipate that many future studies will employ this model to define the mechanisms by which *Bifidobacterium* species regulate goblet cells and interact with intestinal mucus.

Although there are still large gaps in the field, the wealth of literature allows us to make some key observations on conserved bifidobacteria functions, such as mucus binding, suppression of inflammation-driven goblet cell depletion, and elevation of MUC2. Understanding the interaction between *Bifidobacterium* and the intestinal mucus layer is imperative for unraveling the mechanisms underlying their beneficial effects. With this knowledge, there is immense potential for developing targeted therapeutic interventions.

## DECLARATIONS

### Copyright

© The Author(s) 2023.
